# Supplementary material for: Revealing polymerisation defects and formation mechanisms in aldol condensation for conjugated polymers via high-resolution molecular imaging
Source: Nat Commun. 2025 Jul 31;16:7031. doi: 10.1038/s41467-025-62221-y (PMC12314020; doi:10.1038/s41467-025-62221-y)
Supplement: Supplementary file 1 — Supplementary Information [file 41467_2025_62221_MOESM1_ESM.pdf]

SUPPLEMENTARY INFORMATION

Revealing polymerisation defects and formation mechanisms in aldol condensation for conjugated polymers via high-resolution molecular imaging

Table of Contents

1. Materials and methods 2
2. Polymer synthesis 3
3. C=O / CH2 interconversion via hydride transfer 7
4. STM characterisation 8
   4.1 Fitting of the STM images 8
   4.2 Polymer **1** (NN1).... 9
   4.3 Polymer **2** 12
   4.4 Polymer **3** 14
   4.5 Polymer **4** 16
   4.6 Polymer **5** 19
5. NMR characterisation of polymer **4** 23

1. Materials and methods

The generic aldol condensation steps involved in the synthesis of polymers **1**-**5**[1,2,3,4] are shown in Fig. S1. It should be noted that while aldol condensation is used to directly polymerise the comonomers for **1**-**4**, it is only used to make the TBDOPV monomer in the case of **5**, while the polymer is synthesised via Stille polymerisation with a 2,5-bis(trimethylstannyl)thiophene co-monomer[5], as shown in Fig. S2. For more information about the synthetic details, please refer to Section 2.

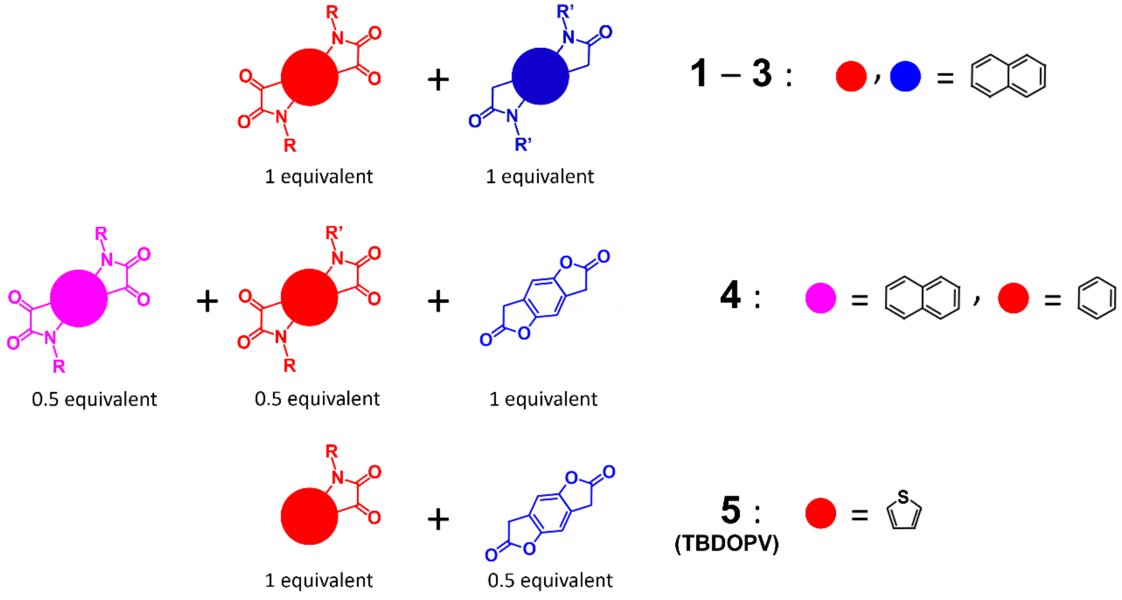

Figure S1. Generic scheme of the aldol condensation reactions employed in the preparation of polymers **1**-**5**. It should be noted that for polymer **5**, the illustrated aldol condensation step is used to synthesise the TBDOPV comonomer, which is subsequently reacted with a 2,5-bis(trimethylstannyl)thiophene co-monomer via Stille polymerisation (shown in Fig. S2).

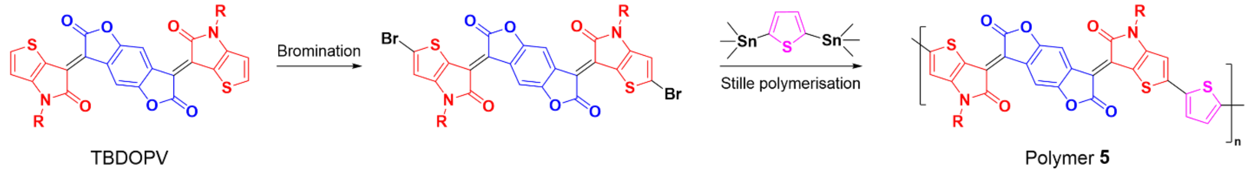

Figure S2. Polymerisation process of polymer **5**. It involves first the bromination of the TBDOPV comonomer, which is subsequently reacted with a 2,5-bis(trimethylstannyl)thiophene co-monomer via Stille polymerisation.

As described in the Methods Section of the main paper, the scanning tunnelling microscopy (STM) experiments on the four polymers **2-5** characterised in this work (Figs. 1b-1e in the main paper) were performed in an ultra-high vacuum (UHV) low temperature (LT) STM system (CreaTec Fischer & Co. GmbH) with a base pressure of 6×10^{-11} mbar at a temperature of −196 °C obtained with a liquid nitrogen bath cryostat. The STM analysis of **1** (Fig. 1 in the main paper) was carried out using a variable temperature (VT) STM system (SPECS Aarhus 150) cooled to −140 °C by a liquid nitrogen flow cryostat. Electrochemically etched tungsten tips were used and prepared by indentation into the metallic surface. Images were acquired in constant current mode with the bias voltage applied to the sample. The metallic substrates underwent a cleaning and preparation procedure involving various cycles of Ar^+ sputtering an annealing, similar to what was done for the LT-STM measurements (see Methods Section in the main paper). Subsequently, the polymer was deposited onto an atomically clean and flat Ag(111) surface held at room temperature by employing the same electrospray deposition (ESD) technique that was used for the four other polymers discussed in this paper.

^{1}H nuclear magnetic resonance (NMR) spectra were recorded on Bruker Avance III HD 400 or 500 MHz spectrometers.

2. Polymer synthesis

Polymer **1** (Supplementary Information (SI) of Ref. [1]):

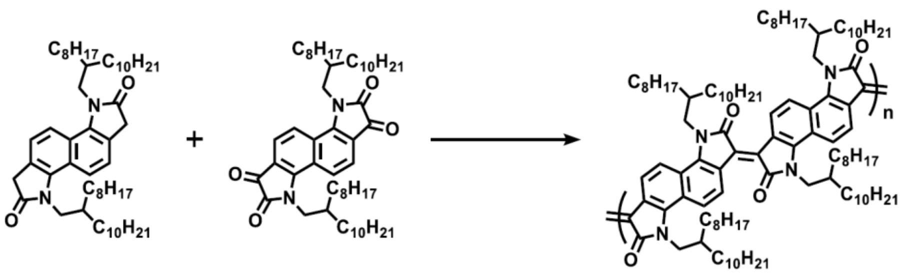

(N-(2-octyltetradecyl))-napthalene bisoxindole (41.80 mg, 0.0523mmol), (N-(2- octyltetradecyl))-napthalene bisisatin (43.26mg, 0.0523mmol) and PTSA monohydrate (4 mg, 0.3eq) were placed into a dry 2mL microwave vial which was capped and evacuated with argon. 0.5mL of degassed

dry toluene was added and the mixture was heated at 120 °C for 2 hours to give a purple solid. Chlorobenzene was added and the polymer was precipitated into methanol. Successive soxhlet extractions with methanol, acetone, hexane and finally DCM gave a single major polymer fraction, which was reduced to minimum volume and precipitated into methanol to give 47 mg of a purple solid (56% yield). GPC (chlorobenzene, 80 °C): Mn 214 kDa, Mw 677 kDa.

Polymer **2** (SI of Ref. [1])

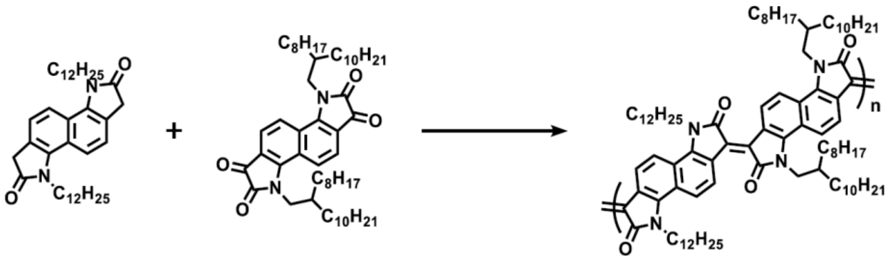

3,8-didodecyl-3,8-dihydroindolo[7,6-g]indole-1,2,6,7-tetraone (66.37mg, 0.08 mmol), 3,8-didodecyl-1,3,6,8-tetrahydroindolo[7,6-g]indole-2,7-dione (46.12 mg, 0.08 mmol) and ptoluene sulfonic acid monohydrate (4.0 mg, 0.02 mmol) were loaded into a dry vial and the vial purged and backfilled with argon three times. Pre-degassed anhydrous toluene (1.5 mL) was injected into the flask and heated at 120 °C for 12 hours, Soxhlet with chlorobenzene gave purple polymer 102 mg, 92% yield. GPC (chlorobenzene, 80 °C): Mn 134 kDa, Mw 538 kDa. ^{1}H NMR (d2-TCE, 403K, 400Hz): δ= 9.36-8.94 ppm (broad), 8.13-7.44 ppm (broad), 4.77-4.03 ppm (broad), 2.44-0.52 ppm (broad).

Polymer **3** (SI of Ref. [2]):

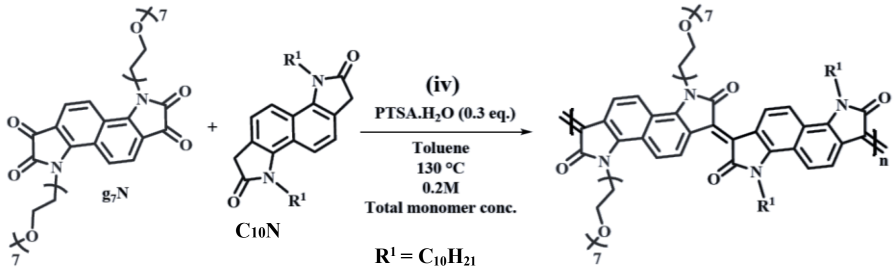

An oven dried 10 mL microwave vial was charged with monomer g_7N (1.0 eq.), monomer $C_{10}N$ (1.0 eq.) and p-toluenesulfonic acid monohydrate (0.3 eq.). The cap was sealed, the vial was then purged with nitrogen for 10 minutes. Degassed anhydrous toluene (3.5 mL) was added, the vial was placed into a pre-heated oil bath (120 °C) and stirred for 22 hours. Upon cooling to room temperature, the dark purple solution was precipitated into 80 mL of methanol, filtered into a thimble, and purified by successive Soxhlet extraction with methanol, acetone, hexane and chloroform. The chloroform fraction was collected and reduced under vacuum, the polymer was re-precipitated into methanol, filtered and dried to obtain a dark purple film.

Polymer **4**:

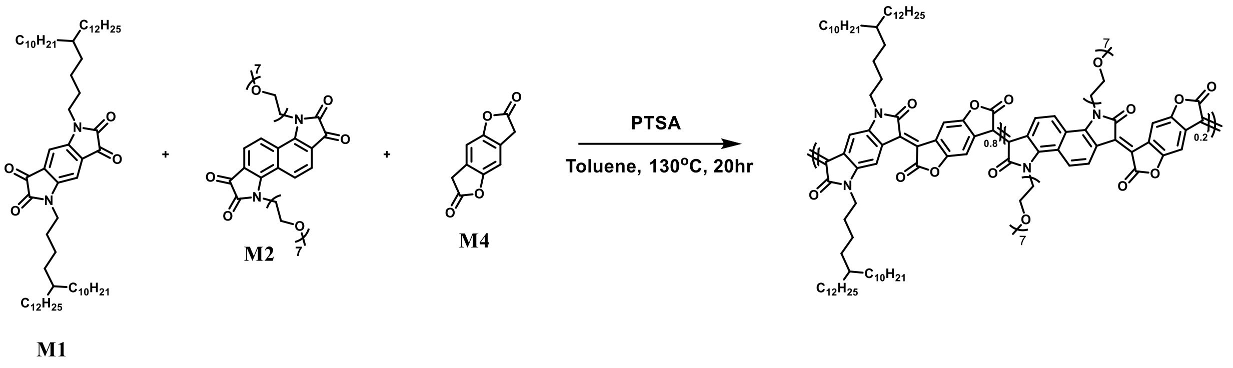

A microwave vial was charged with M4 (37.1 mg, 0.195 mmol), M1 (95 mg, 0.0976 mmol), M2 (88.8 mg, 0.0975 mmol) and *p*-toluene sulfonic acid monohydrate (6 mg, 0.0585 mmol). Anhydrous toluene (1.5 mL) was injected. The resulting solution was purged with N_2 for 30 min and the reaction was heated to 130 °C for 20 hours. The reaction mixture changed colour from dark purple to dark blue over the polymerisation period. The crude polymer was precipitated in methanol and purified by Soxhlet extraction with methanol, acetone, hexane and finally chloroform. The chloroform fraction was collected and the solvent was removed under vacuum. Afterwards, the polymer was precipitated into methanol, filtered and dried. Yield: 85 mg, 45 % dark blue solid. GPC (Chloroform, 40 °C): M_n 18 kDa, M_w 33 kDa. ^{1}H NMR (400 MHz, *o*-$C_6D_4Cl_2$-*d4*) δ 8.11-9.50 ppm (aromatic peaks, very broad), 3.80-3.15 ppm (glycol peaks, broad), 1.74-0.81 ppm (alkyl peaks, broad).

Polymer **5**:

Aldol condensation to synthesise TBDOPV monomer (SI of Ref. [4]):

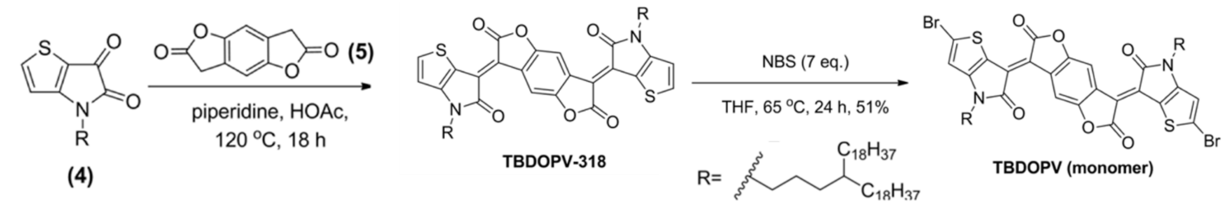

To a solution of M**4** (2.00 mmol) in acetic acid (15 mL), M**5** (190 mg, 1.00 mmol) and piperidine (0.20 mmol) was added. The mixture was stirred at 120 ºC under nitrogen atmosphere for 20 h, then cooled to room temperature and filtered. After washed with acetic acid and methanol, the filtration residues were dissolved in $CHCl_3$ (100 mL). After removal of the solvents under reduced pressure, the residue was purified by silica gel chromatography with eluent (PE: $CHCl_3$ = 4:1) to give a dark solid.

To a solution of TBDOPV-318 (581 mg, 0.367 mmol) in THF (100 mL), NBS (457 mg, 2.57 mmol) was added. The mixture was stirred at 65 ºC under nitrogen atmosphere for 24 h. After removal of the solvents under reduced pressure, the residue was dissolved in CHCl3 (100 mL) and then washed with water and brine, and dried over with Na_2SO_4. After removal of the solvents under reduced pressure, the residue was purified by silica gel chromatography with eluent (PE : $CHCl_3$ = 1:8) to give a dark solid.

HPLC purification using a preparation-scale size exclusion chromatography with chloroform as eluent was carried out to guarantee the purity of the TBDOPV monomer before polymerisation.

Stille polymerisation to synthesise polymer **5** (SI of ref [5]):

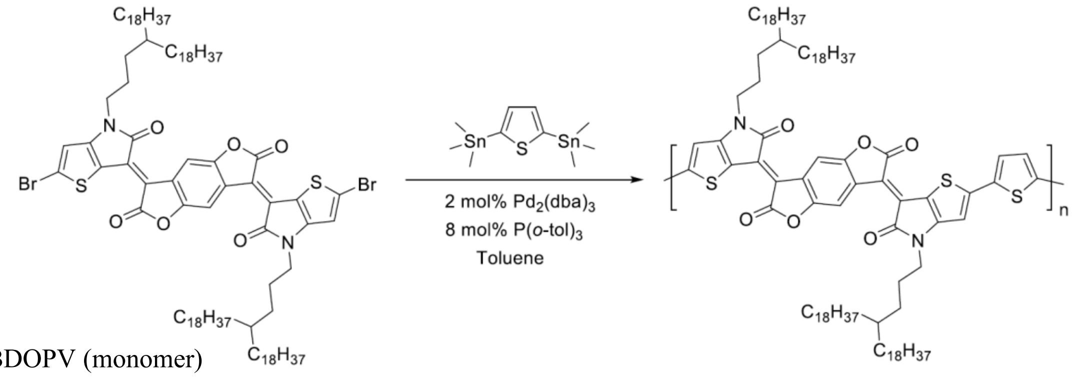

TBDOPV (monomer)

To a Schlenk tube TBDOPV-318 (60.00 mg, 0.03448 mmol), 2,5-bis(trimethylstannyl)thiophene (14.13 mg, 0.03448 mmol), $Pd_2(dba)_3$ (0.65 mg, 2 mol%), P(*o*-tol)$_3$ (0.85 mg, 8 mol%), and toluene (12 mL) were added under nitrogen atmosphere. After three freezepump-thaw cycles, the mixture was stirred at 120 ºC for 24 h, and then diethylphenylazothioformamide (10 mg) was added. The mixture was stirred for 1 h to remove any residual catalyst before being precipitated into methanol (200 mL). The precipitate was filtered through a PTFE filter and purified via Soxhlet extraction for 8 h with methanol, 8 h with acetone, 12 h with hexane, and finally was collected with chloroform (12 h). The chloroform solution was concentrated by evaporation and then precipitated into methanol (200 mL) and filtered off to afford polymer **5** as dark solids. Elemental Anal. Calcd for $(C_{106}H_{168}N_2O_6S_3)_n$: C, 76.57; H, 10.18; N, 1.68; Found: C, 76.32; H, 10.23; N, 1.57.

3. C=O / CH2 interconversion via hydride transfer

The C=O / CH_2 interconversion via hydride transfer is possible in the acidic medium of the aldol reaction due to polarisation of the protonated ketone and the subsequent donation of a hydride from the benzylic position of a CH_2 monomer. After addition of water to the cationic intermediate both monomer are alcohols, and then the reaction can either reverse or proceed, either restoring or swapping the CH_2 and C=O functional groups between the comonomers. Such a mechanism has been proposed in the rearrangement of benzopyrenones[6].

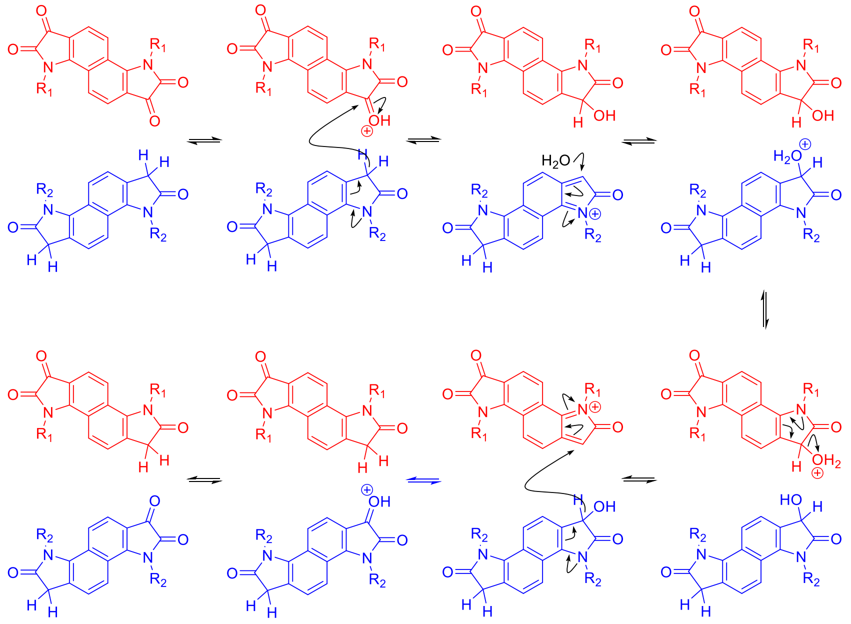

Figure S3. Mechanism proposed for the C=O / CH_2 interconversion via hydride transfer in A and B comonomers, prior to aldol condensation reaction.

4. STM characterisation

4.1 Fitting of the STM images

In order to determine the polymer sequence and to identify the different coupling defects as illustrated in Fig. 5a, the features observed in high-resolution STM images are subjected to a rigorous molecular model fitting procedure, as described in the following. The methodology begins with the geometry optimisation of the molecular models of the individual comonomers (*e.g.*, for polymer **2**, of the A and B comonomers depicted in red and blue, respectively, in Fig. 1) and of the side chains; this optimisation is carried out separately in the Avogadro software by employing the MMFF94 force field. Subsequently, the geometry-optimised molecular models are precisely overlayed onto high-resolution STM images in the LMAPper software[7], in a series of successive steps. The backbones are firstly identified in the STM images as continuous central features, flanked by bright dots that are attributed to the connection points of the side chains to the

backbones (*e.g.*, see large and small oval-shaped bright dots in Fig. S5a, highlighted in red and blue dots in Fig. S5b, respectively).

The actual fitting process commences by superimposing the molecular models of the side chains onto the images, taking the position of the bright dots as a reference. The distinction between different side chains (branched *vs* linear, alkyl *vs* glycol) can be readily identified based on their shapes and lengths, as well as on the size of the corresponding bright dots in the STM images. Once the positions of the side chains are confirmed, the positioning of the comonomers is determined by ensuring that the two nitrogen atoms on both sides of the comonomers are directly connected to the side chains. For **4** and **5**, the gaps between comonomers with side chains are filled with side chain-free lactone-based moieties, as well as thiophene rings, depending on the available space within the backbone structure. The orientation of the comonomers is ascertained by considering the serpentine motif of the backbone in the STM images[8], as well as the relative position of side chains on both side of the backbones.

The next step involves substituting successive individual comonomers in the above fitted images (*e.g.* A and B for **2**) with the corresponding covalently linked AB units, which had been separately optimised in Avogadro (as shown in Fig. S7). The best fitting of these AB units ensured that the angles and orientations of the comonomers within the backbones and the positions of the side chains precisely corresponded to the features in the STM images. This fitting procedure was repeated iteratively, involving consecutive adjustments of the different comonomers, until an optimal match was achieved between the STM images and the molecular models. This allowed us to reliably and reproducibly determine the polymer sequence and the comonomer coupling. Finally, the defect content of the polymers was estimated by quantifying the relative frequency of each type of coupling between comonomers (*e.g.*, see Fig. 5a) on a large set of high-resolution STM images.

4.2 Polymer 1 (NN1)

Figure S4a shows a large scale STM image of **1** deposited by ESD onto Ag(111). While the backbones are mostly straight, which is what would be expected for "regular" c1 *trans* couplings

between the monomers (see Figs. 3a and S7), occasional sharp bends can be observed. Some of these are marked by pink and red chevrons in Fig. S4a, corresponding to kinks in the backbones with angles of (130 ± 5)° and (80 ± 5)°, respectively. The former are compatible with a c2 *cis* coupling defect (see Figs. 3a, S7 and S4b), while the latter would be expected for two successive and spatially close *cis* coupling defects, as shown in Fig. S4b.

We note that rotation around C=C double bonds is energetically highly unfavourable, with substantial energy barriers (*e.g.*, approximately 65 kcal/mol for the *cis–trans* isomerisation of but-2-ene)[9]. These barriers are significantly greater than the kinetic energy imparted to the polymer chains during ESD deposition. Therefore, we can confidently exclude the possibility that the observed coupling defects arise during the ESD process; instead, they must originate from processes occurring during polymer synthesis.

Without access to high-resolution images, in the case of **1** it was not possible to perform the detailed analysis that was done for the polymers **2**-**5** in the main paper. However, an initial and basic statistical analysis of the overall frequency of monomer coupling defects could be carried out by taking the simplifying assumption that all observed kinks represent single defects (the ~80° kinks being quite rare). Specifically, a large number of STM images was analysed by superposing segmented lines onto individual polymer molecules (black lines in Fig. S4a) and determining their total length and the length of all straight segments within a polymer. Based on this, two distributions were determined, the polymer total length distribution and the length distribution of straight segments in between successive defective couplings. These are shown in Figs. S4c and S4d, respectively, as a function of the number of composing monomers and are obtained from the original length distributions by considering that the repeat unit of **1** has a length of 0.91 nm. From these two distributions, the following numbers can be obtained: N_p, the total number of analysed polymers, N_s, the total number of straight segments and N_m, the total number of monomers. The total number of bonds, N_b, is simply given by

$$N_b = N_m - N_p$$

while the total number of defective bonds (kinks), N_d, is given by

$$N_d = N_s - N_p$$

Thus, the relative frequency of *cis*-defects is obtained as

$$f_{cis} = \frac{N_d}{N_b} = 9.1\%$$

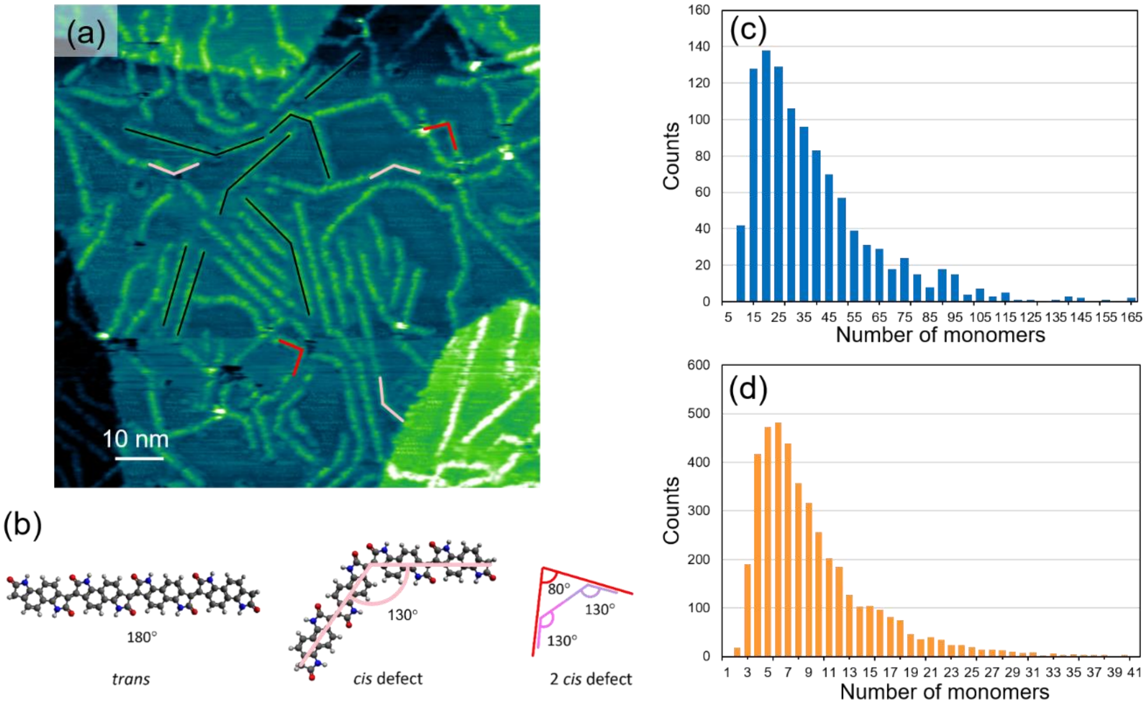

Figure S4. (a) Large scale STM image of **1** vacuum deposited by ESD onto a Ag(111) surface ($V = -2.0$ V, $I = 30$ pA). The black lines superimposed on individual polymer strands show examples of how the length of individual polymers and of the straight backbone segment in between successive kinks were evaluated. When performed on a large number of images, their frequency analysis is at the basis of the distributions in (c) and (d), respectively. A few individual kinks and pairs of closely consecutive kinks are marked by pink and red chevrons, respectively. (b) Molecular models of backbone segments in *trans* and *cis* configurations, with the corresponding kink angles and schematic representation of two closely consecutive kinks. (c) Length distribution of polymer strands. (d) Length distribution of straight segments in between successive defective couplings.

4.3 Polymer 2

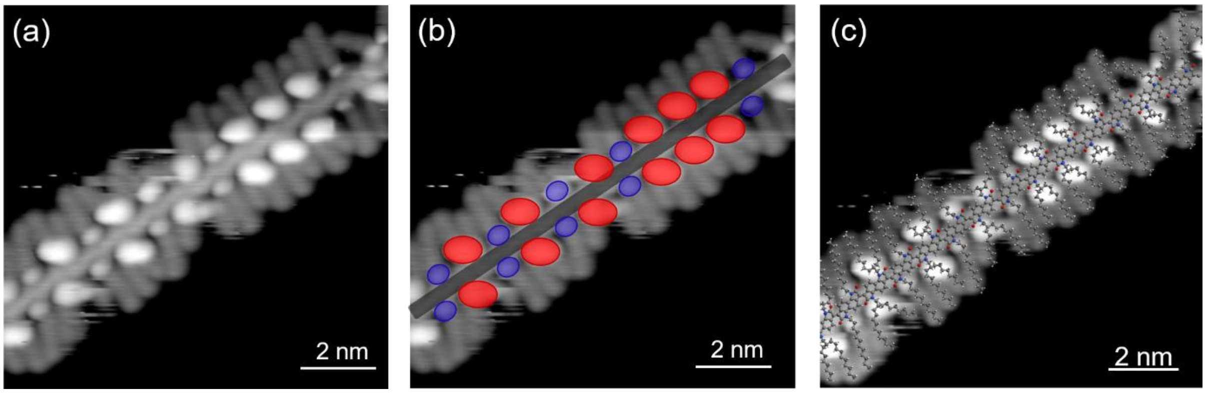

Figure S5. (a) High-resolution STM image of an isolated **2** polymer strand (10 nm × 10 nm, $V = 149$ mV, $I = 120$ pA). (b) The central region representing the backbone is highlighted in grey, the large oval-shaped bright dots connecting the backbone to the $C_2C_{8;10}$ branched side chains are highlighted in red, and the small oval-shaped bright dots connecting the backbone to the C_{12} linear side chains are highlighted in blue. (c) A scaled molecular model of the polymer is overlayed onto the STM image as in Fig. 2c.

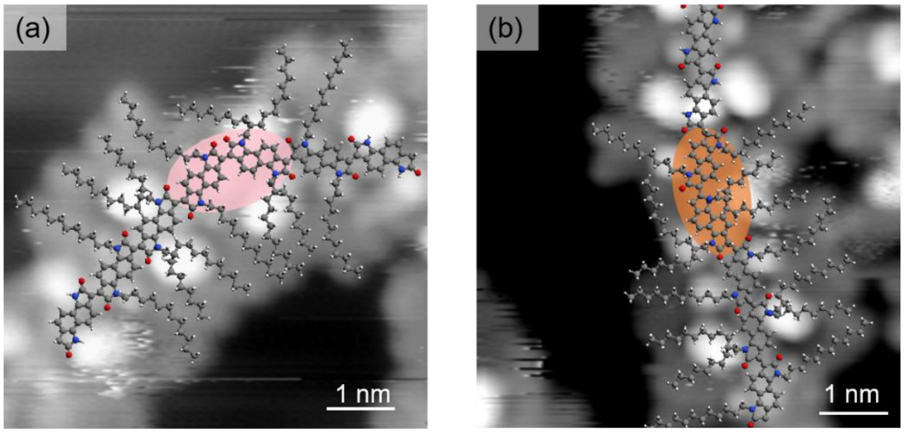

c2 **c3**

Figure S6. High-resolution STM images of **2** showing the formation of kinks in the backbone. (a) shows a c2 coupling, (b) a c3 coupling, as illustrated in Fig. 5a. Scaled molecular models of the polymer are overlayed to the STM image. The kinks are marked by pink and orange ovals in figures (a) and (b), respectively. Image parameters are (a): 5 nm × 5 nm, $V = 164$ mV, $I = 95$ pA; (b): 5 nm × 5nm, $V = 219$ mV, $I = 90$ pA.

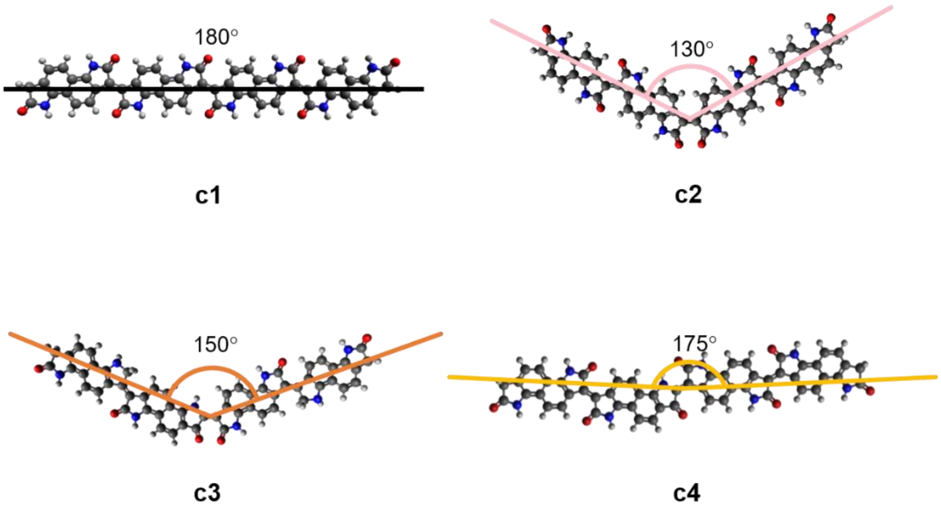

Figure S7. Molecular models of c1 to c4 couplings as illustrated in Fig. 5a, geometry-optimised by the MMFF94 force field in Avogadro. The angle of the kink formed by different couplings is indicated.

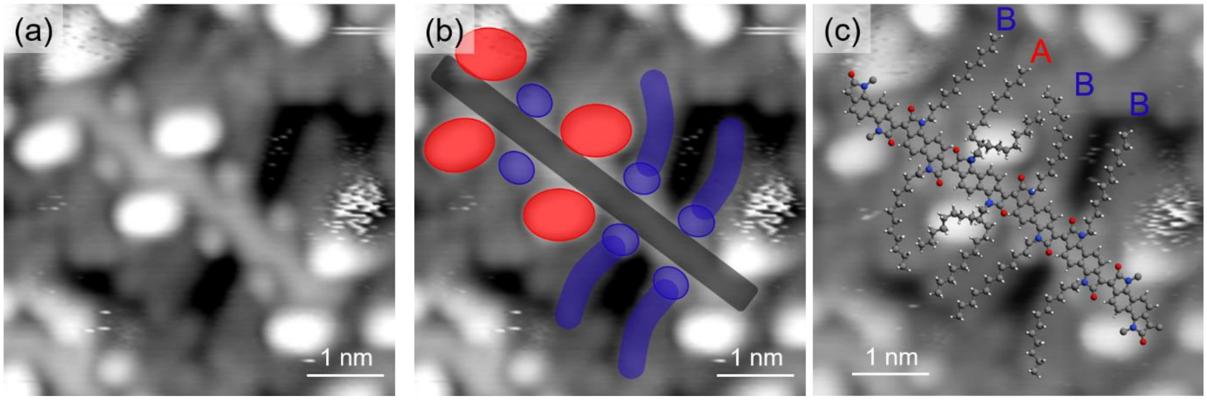

Figure S8. High-resolution STM image of **2** showing defects in the polymer sequence with two successive B units (5 nm × 5 nm, V = 121 mV, I = 90 pA) in a straight stretch of the polymer. A schematic representation of the polymer is superposed on the STM image in (b), highlighting the backbone in grey, the large bright dots connecting the backbone to branched side chains by red ovals, the small bright dots connecting the backbone to linear side chains by blue ovals and the linear C_{12} side chains by blue thick lines. (c) A scaled molecular model of the polymer is overlayed onto the STM image. Letters A and B refer to the two comonomers of polymer **2** defined in Fig. 1.

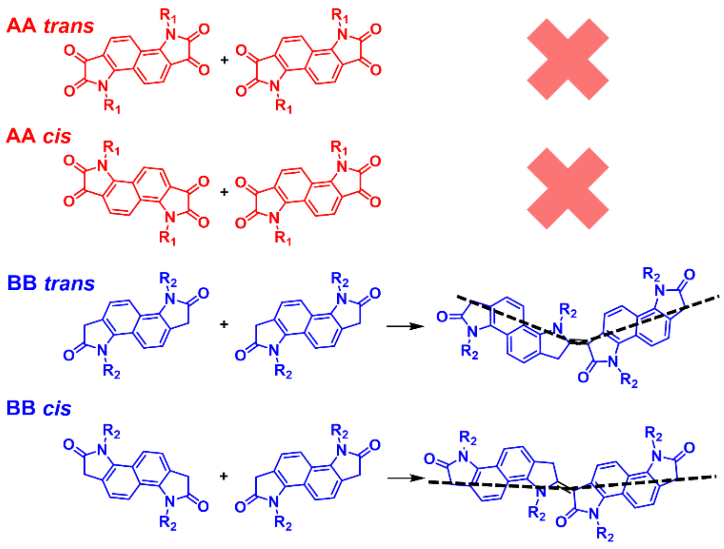

Figure S9. Illustration of potential homocoupling reaction for A and B comonomers. A comonomers cannot react with each other by aldol condensation. BB homocouplings, both in *cis* and *trans* configurations, will always lead to the formation of kinks, as illustrated by the black dashed lines on top of the molecular structure. Moreover, a *cis* BB homocoupling would be characterised by having two successive side chains on the same side of the backbone.

4.4 Polymer 3

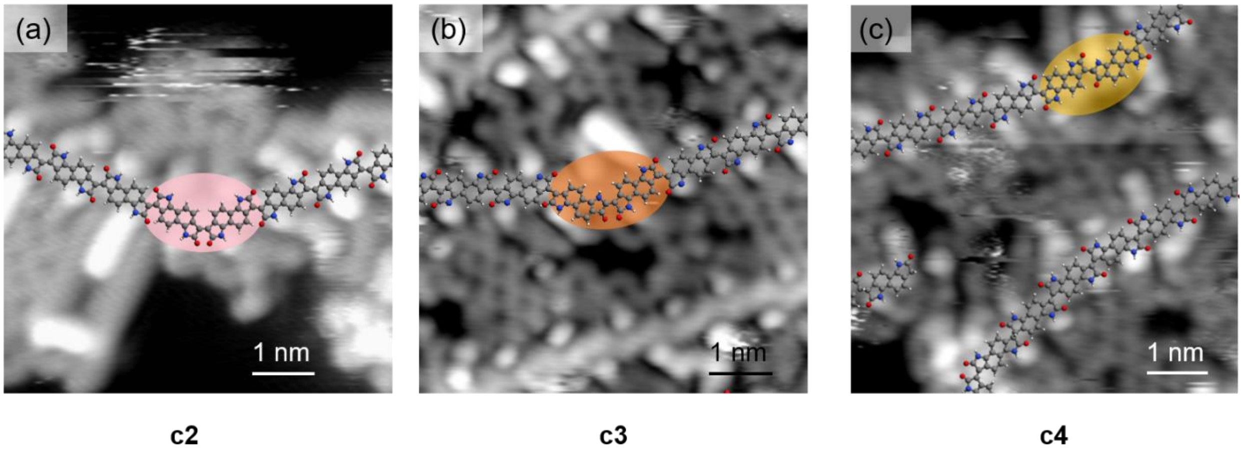

Figure S10. High-resolution STM images of **3** showing the formation of kinks in the backbone caused by (a) c2 (5 nm × 5 nm, V = 247 mV, I = 140 pA), (b) c3 (5 nm × 5 nm, V = 209 mV, I = 150 pA) and (c) c4 (5 nm × 5 nm, V = 176 mV, I = 110 pA) couplings as defined in Fig. 5a. Scaled molecular models of the polymer backbone are overlayed onto the STM images (no side chains are included in the models). The kinks are marked by pink, orange and yellow ovals in images (a), (b) and (c), respectively.

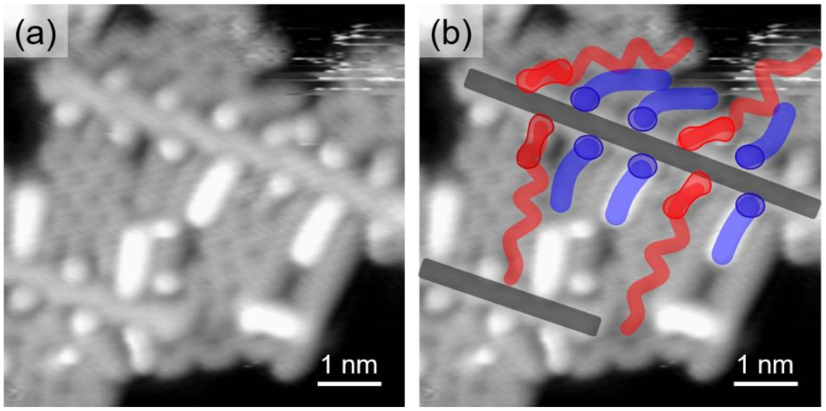

Figure S11. High-resolution STM image of **3** showing EG and alkyl side chains protruding from two straight backbones oriented parallel to each other (5 nm × 5 nm, V = 247 mV, I = 140 pA). The backbones are highlighted in grey in (b), and the initial sections of the side chains appearing as small-oval and large-dumbbell features are highlighted in blue and red, respectively. The EG and alkyl side chains are marked by wiggled red lines and linear blue lines, respectively.

Table S1. 95% confidence intervals (CI) corresponding to the values reported in Table 1 of the main paper for polymers **2** and **3**. These intervals are Goodman multinomial 95% CI for all the individual coupling or sequence entries and binomial 95% CI for the sums. Polymers **2** and **3** are marked in bold format.

Coupling	**2**	**3**
c2	1.5-4.6%	2.1-6.2%
c3	0.8-3.3%	0.2-2.4%
c4	0-0.9%	0-1.8%
Sum	3.0-6.2%	3.1-7.0%
c1	93.3-97.3%	92.4-97.1%

Sequence	**2**	**3**
AA	0.6-2.9%	1.6-5.2%
BB	1.1-3.8%	9.0-15.7%
Sum	2.3-5.2%	12.7-18.2%
AB	94.4-97.9%	81.1-88.4%

Monomer	**2**	**3**
A	48.1-55.1%	43.3-51.0%
B	44.9-51.9%	49.0-56.7%

4.5 Polymer 4

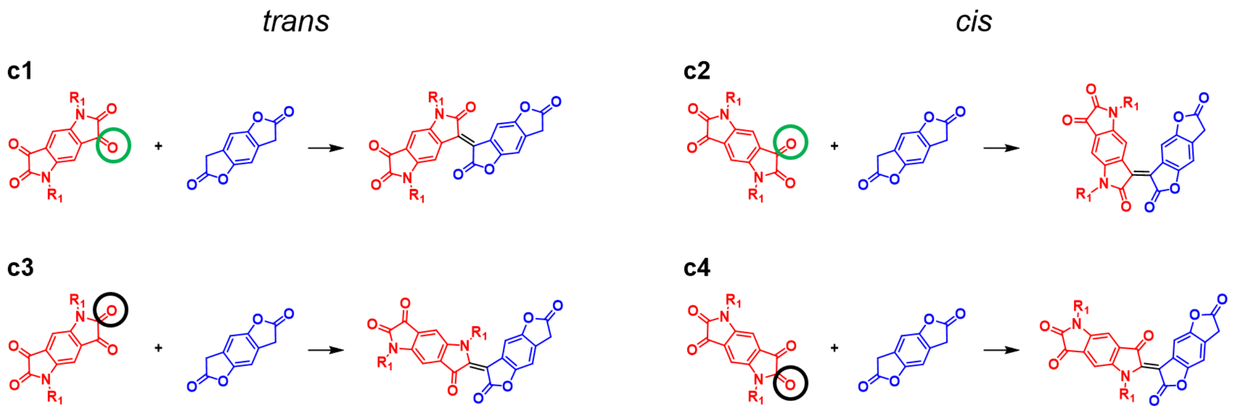

Figure S12. Possible reaction pathways for coupling defects between the A lactam-based and the B lactone-based comonomers in **4** leading to the formation of kinks in the polymer backbone. The β and α carbonyl positions of the pyrrole ring where the reaction takes place are circled in green and black, respectively. A and B comonomers are drawn in red and blue, respectively.

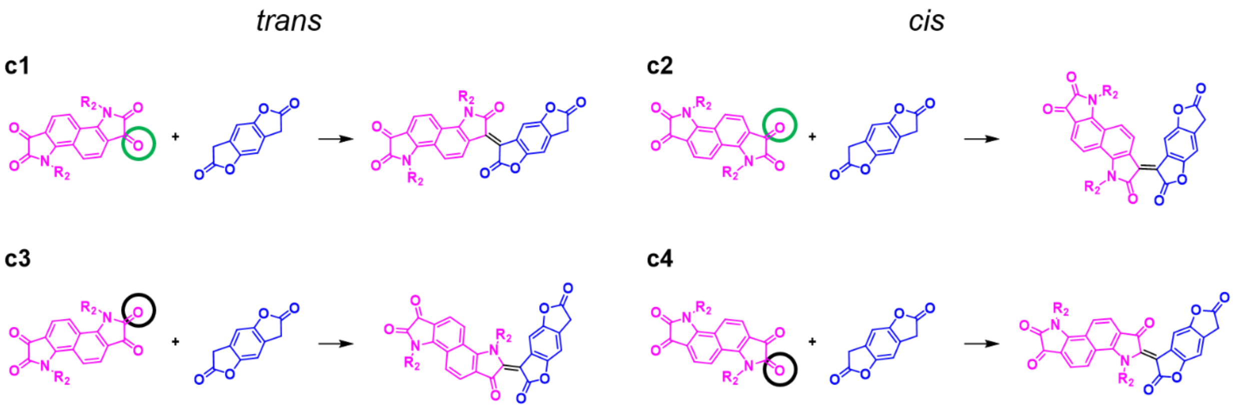

Figure S13. Possible reaction pathways for coupling defects between the C lactam-based and the B lactone-based comonomers in **4** leading to the formation of kinks in the polymer backbone. The β and α carbonyl positions of the pyrrole ring where the reaction takes place are circled in green and black, respectively. B and C comonomers are drawn in blue and magenta, respectively.

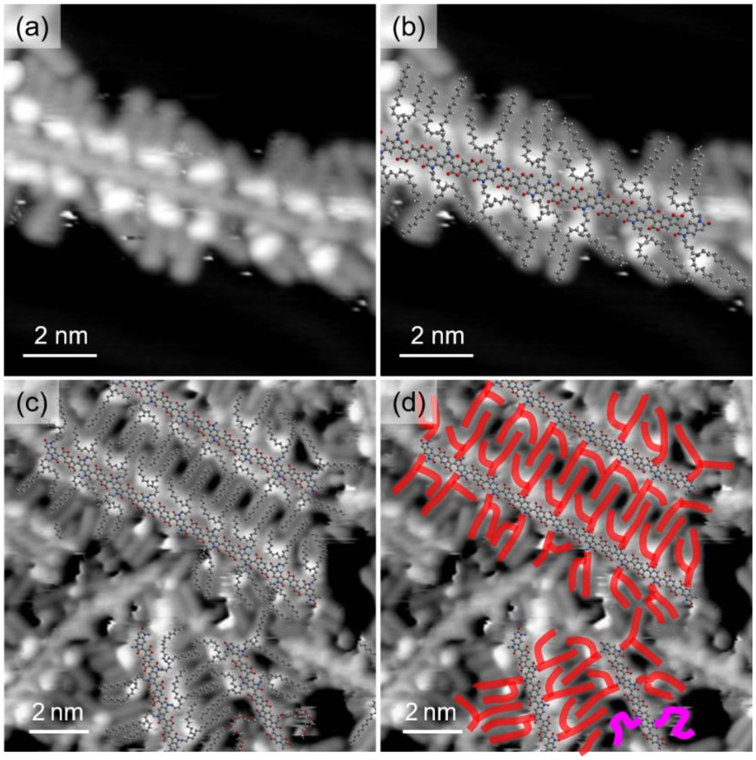

Figure S14. (a) High-resolution STM image of an isolated **4** polymer showing only branched alkyl side chains protruding from a straight backbone (10 nm × 10 nm, $V = 350$ mV, $I = 78$ pA). (b) A scaled molecular model of the polymer is superposed onto the same STM image as in (a). (c) High-resolution STM image of a high coverage area of **4** (same as in Fig. 7a). (d) The side chains are indicated by red and magenta lines for branched alkyl and EG side chains, respectively.

The measured separation between two successive branched side chains in polymer **4** is (13.5 ± 0.5) Å, which is in good agreement with the expected length (13.7 Å, see Fig. S15) of an isatin unit A followed by a side chain-free lactone unit B. A statistical analysis conducted over several dozen STM images acquired in various locations on the surface indicates that **4** exhibits an overwhelming majority of 80% AB couplings compared to only 20% BC couplings (see Table S2). The same analysis also indicates that the distribution of comonomers in **4** is composed by 43% of A units, 47% of B units, and only 10% of C comonomers. This is significantly different from the molar ratio of 25%:50%:25% between A, B, and C comonomers initially introduced in the reaction solution[3].

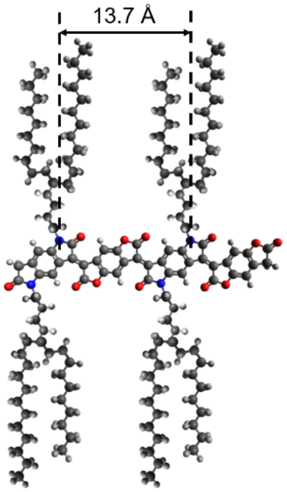

Figure S15. Molecular model of an AB coupling (see Fig. 1 for the definition of the A and B comonomers in polymer **4**) optimised by the MMFF94 force field in Avogadro. The polymer periodicity is indicated by a black double arrow.

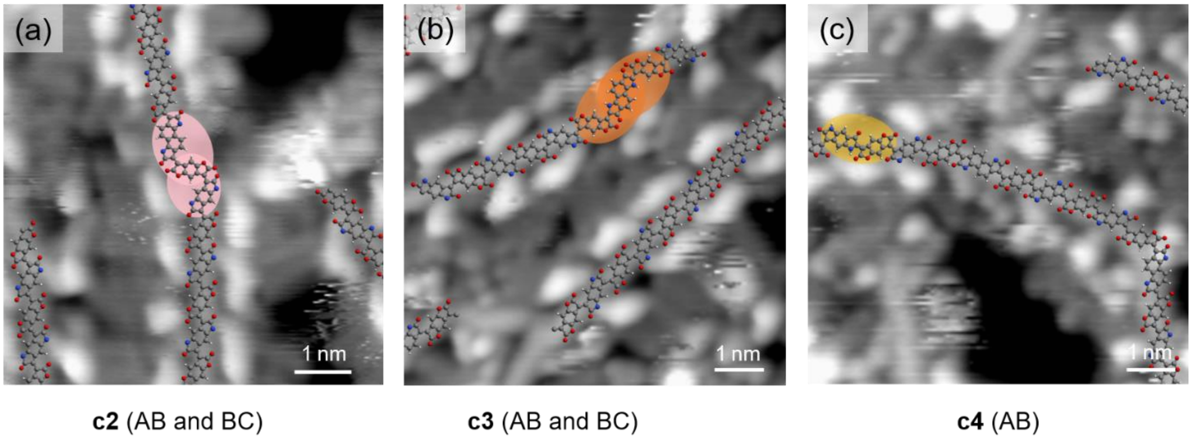

Figure S16. High-resolution STM images of **4** showing the formation of kinks in the backbone caused by (a) successive c2 (5 nm × 5 nm, V = 573 mV, I = 78 pA), (b) successive c3 (5 nm × 5 nm, V = 213 mV, I = 120 pA) couplings between AB and BC comonomers and (c) c4 (5 nm × 5 nm, V = 552 mV, I = 67 pA) coupling between AB comonomers, as illustrated in Figs. S12 and S13. Scaled molecular models of the polymer backbone are overlayed onto the STM images. The kinks are marked by pink, orange and yellow ovals in images (a), (b) and (c), respectively.

Table S2. Backbone defect frequencies for polymer **4**. While c1 is the correct *trans* coupling, c2-c4 are defective couplings, as illustrated in Fig. 5a. The relative frequencies were calculated with respect to the total amount of analysed coupling configurations (left). For the table on the right, A, B and C represent the three comonomers of **4**, as shown in Fig. 1. The 95% CI are Goodman multinomial 95% CI for all the individual coupling or sequence entries and binomial 95% CI for the sums.

Coupling	Count	Frequency	95% CI
c2	24	2.0%	1.1-3.4%
c3	19	1.6%	0.8-2.9%
c4	2	0.2%	0-0.9%
Sum	45	3.7%	2.8-5.1%
c1	1172	96.3%	94.4-97.6%

Sequence	Count	Frequency	95% CI
AB	963	80.3%	77.8-82.4%
BC	237	19.7%	17.6-22.2%

Monomer	Count	Frequency	95% CI
A	591	42.8%	39.5-46.1%
B	650	47.1%	43.7-50.4%
C	140	10.1%	8.3-12.3%

4.6 Polymer 5

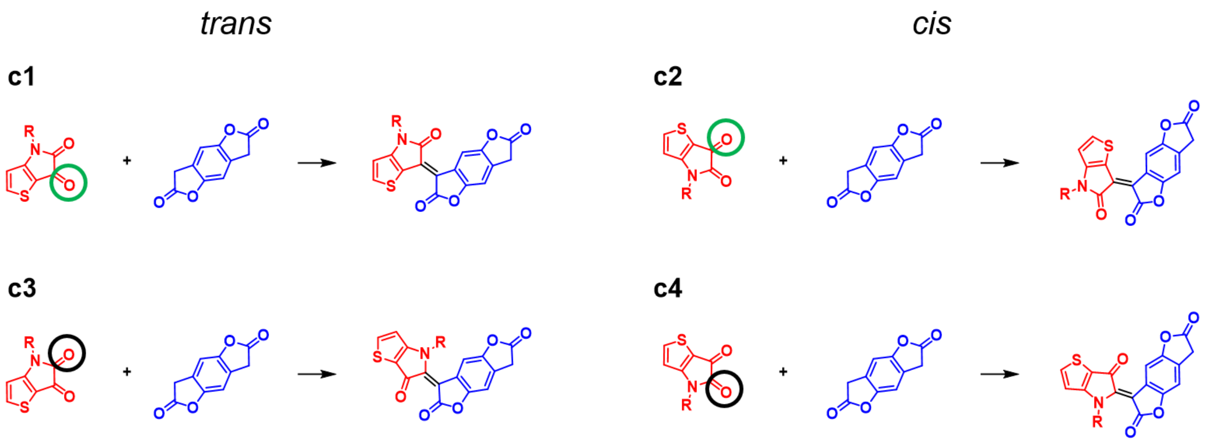

Figure S17. Possible reaction pathways for coupling defects between the A lactam-based and the B lactone-based small molecules reacted by aldol condensation for the construction of the TBDOPV comonomer, leading to the formation of kinks in the polymer backbone. For conciseness, only one of the two A lactam-based molecules in the TBDOPV comonomer is shown here, but the defective reactions involving the other one are expected to be analogous. The β and α carbonyl positions of the pyrrole ring where the reaction takes place are circled in green and black, respectively. A and B molecules are drawn in red and blue, respectively.

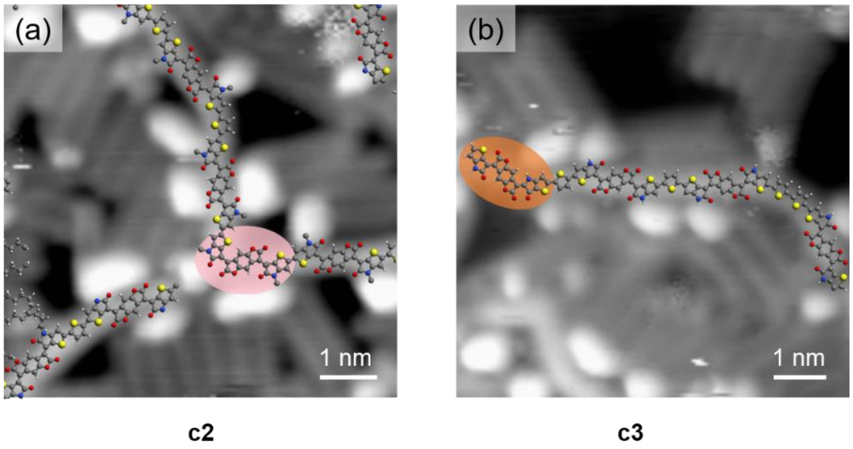

Figure S18. High-resolution STM images of **5** showing the formation of kinks in the backbone caused by (a) c2 (5 nm × 5 nm, V = 247 mV, I = 140 pA) and (b) c3 couplings (5 nm × 5 nm, V = 149 mV, I = 120 pA) as illustrated in Fig. S17. Scaled molecular models of the polymer backbone are overlayed onto the STM images. The c2 and c3 kinks are marked by pink and orange ovals, respectively.

Table S3. Coupling frequencies for polymer **5**. While c1 is the correct *trans* coupling, c2-c4 are defective couplings, as illustrated in Fig. 5a. The relative frequencies were calculated with respect to the total amount of analysed coupling configurations (left). The 95% CI are Goodman multinomial 95% CI for all the individual coupling entries and binomial 95% CI for the sums.

Coupling	Count	Frequency	95% CI
c2	5	0.8%	0.2-2.6%
c3	3	0.5%	0.1-2.1%
c4	0	0.0%	0-1.3%
Sum	8	1.2%	0.5-2.6%
c1	634	98.8%	96.8-99.6%

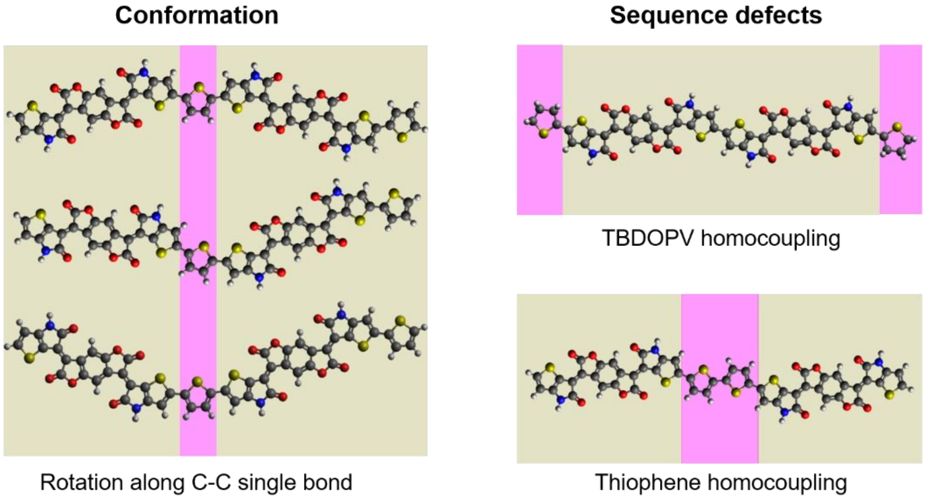

Figure S19. Schematic representation of conformational and sequence defects in **5**. The conformational defects are due to the rotation around C-C single bonds formed by Stille polymerisation. Homocoupling defects can happen between TBDOPV comonomers as well as between thiophene rings during Stille polymerisation.

Homocoupling defects generated during the Stille reaction cycle were observed for **5,** both between TBDOPV comonomers (marked by olive rectangles in Fig. S20 and illustrated by model in Fig. S18) and between thiophene units (marked by magenta rectangles in Fig. S20 and illustrated by models in Fig. S19). Finally, the precise fitting of the STM images also revealed a distinct type of backbone kink, different from those associated with coupling defects c2-c4, which occur specifically at the thiophene units. These are caused by 180° rotation around C-C single bonds and are thus identified as conformational defects. Examples of such defects are highlighted by purple rectangles in Fig. S21 and illustrated by models in Fig. S19.

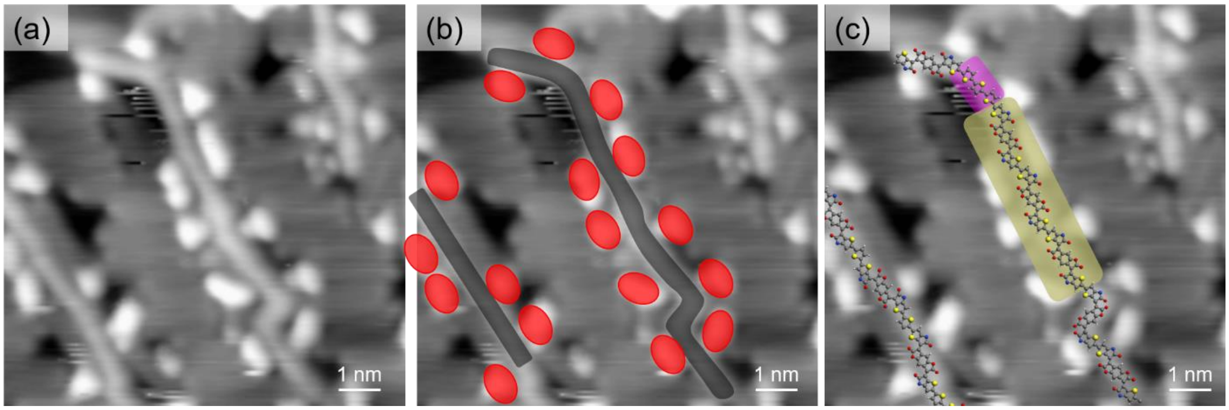

Figure S20. High-resolution STM image of **5** showing homocoupling defects due to Stille polymerisation (5 nm × 5 nm, V = 346 mV, I = 78 pA). In (b) the backbone is highlighted in grey and the initial sections of the branched side chains appearing as large oval-shaped features in (a), are highlighted in red. (c) Scaled molecular models of the polymer backbone are superposed onto the STM image. The structures of TBDOPV and thiophene homocouplings illustrated in Fig. S19 are highlighted by olive and magenta rectangles in (c), respectively.

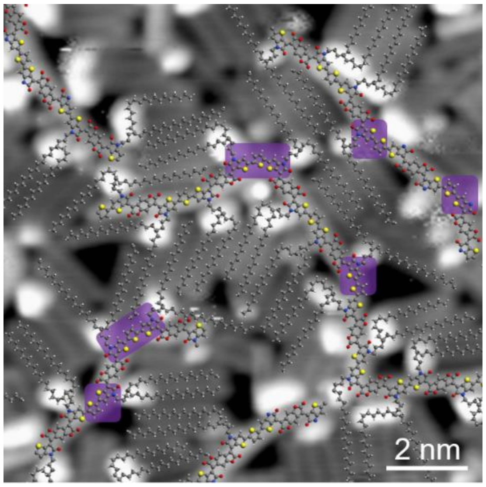

Figure S21. High-resolution STM image of **5** (same as Fig. 7d, 10 nm × 10 nm, V = 247 mV, I = 140 pA) showing conformational defects due to the rotation of C-C single bond as illustrated in Fig. S19. Geometry-optimised molecular models are superposed onto the image.

5. NMR characterisation of polymer 4

Variable temperature (VT) NMR experiments were performed to obtain higher-quality spectra of polymer **4** over a temperature range of 5 to 100 °C in o-$C_6D_4Cl_2$-*d*4 (Figure S22). A notable change in the dynamics of the alkyl region (1.13–1.90 ppm) was observed, including an upfield shift of the most prominent peak from 1.90 ppm to 1.53 ppm. In contrast, the glycol region (3.54–4.50 ppm) showed no significant changes over the same temperature range. Interestingly, at 100 °C, the alkyl peaks became sharper and better resolved. Subsequently, 1400 scans were acquired at 100 °C to integrate the alkyl and glycol regions. As shown in Figure S23, the integration yielded an alkyl:glycol ratio of 0.80:0.20, consistent with the STM results of polymer **4** (see Table S2).

Since water signals can overlap with polymer peaks and potentially lead to inaccurate integrations, we also performed a water suppression NMR experiment. Both the standard at 100 °C and room-temperature water-suppressed ^{1}H NMR spectra gave comparable alkyl-to-glycol ratios, as illustrated in Figures S24 and S25.

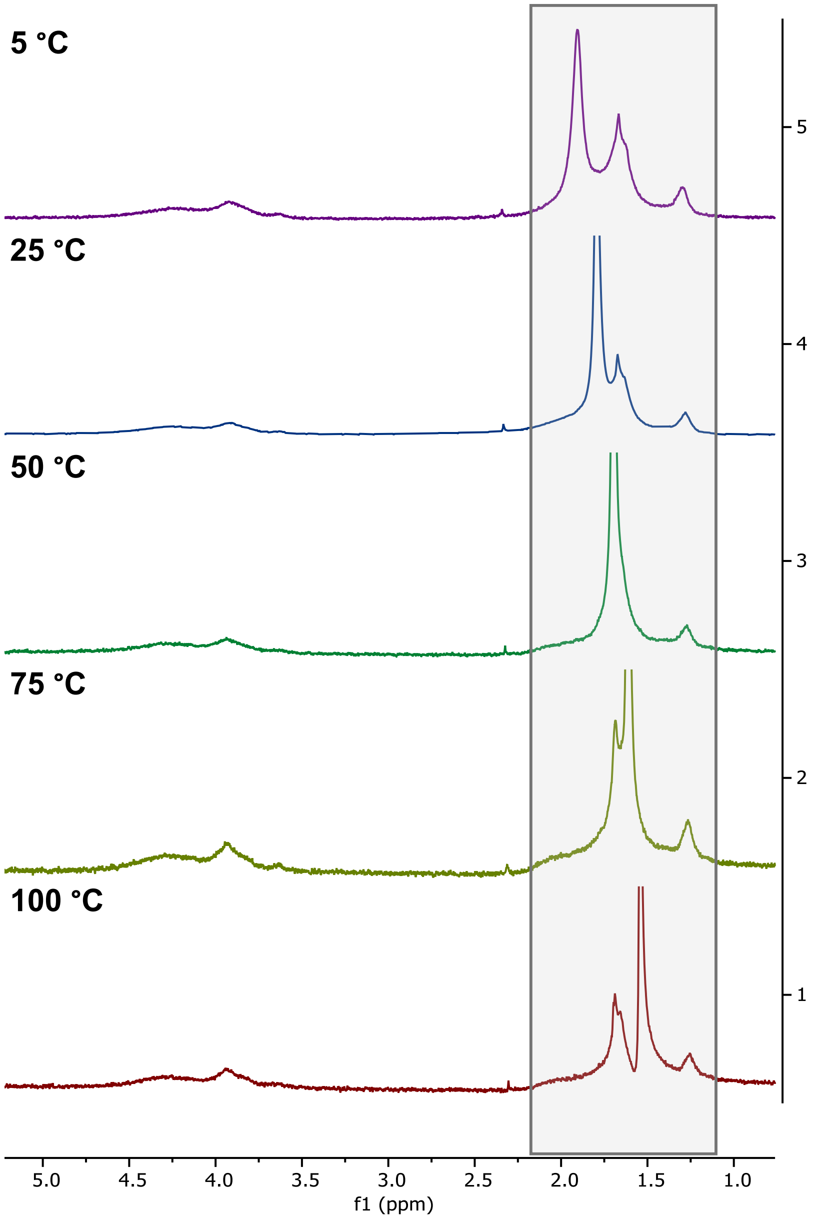

Figure S22. ^{1}H NMR spectra of polymer **4** acquired in o-$C_6D_4Cl_2$-*d*4 at different temperatures.

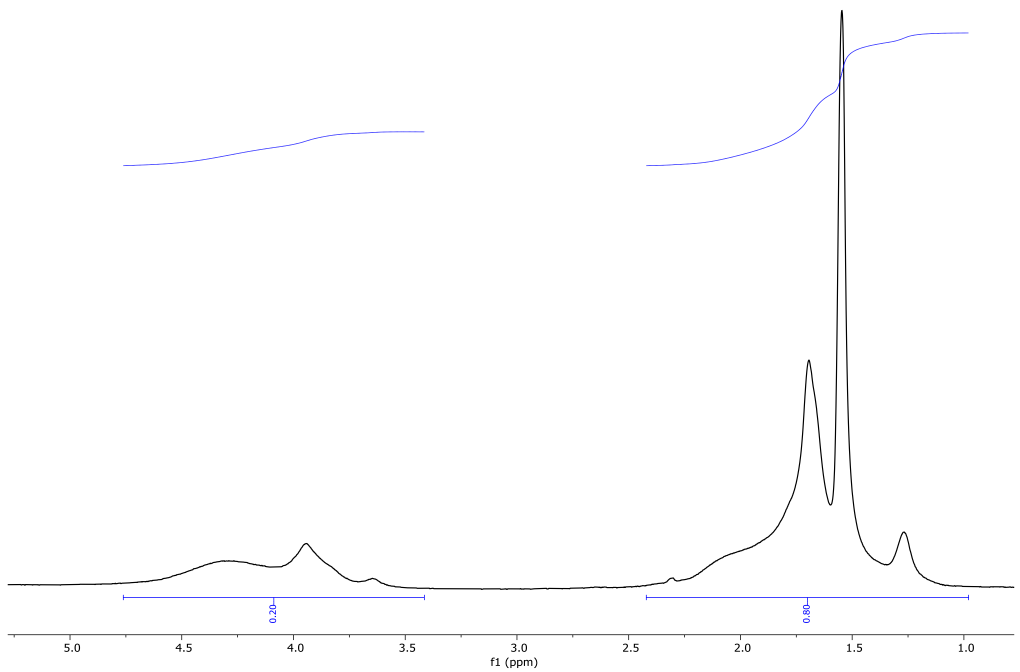

Figure S23. ^{1}H NMR spectrum of polymer **4** acquired in o-$C_6D_4Cl_2$-*d*4 at 100 °C.

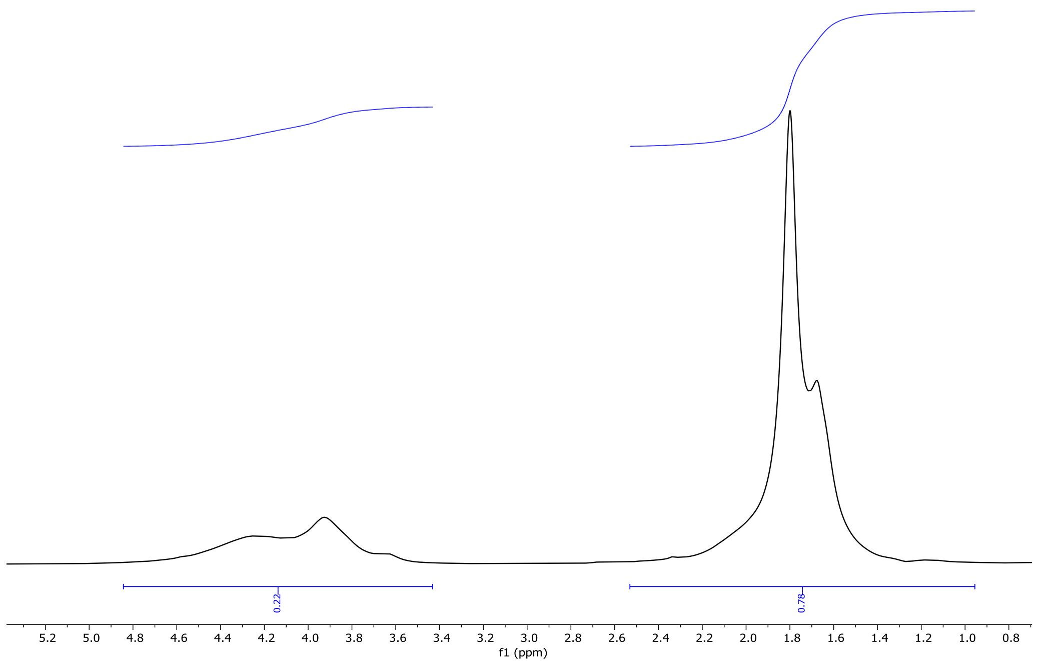

Figure S24. Water suppression ^{1}H NMR spectrum of polymer **4** acquired in o-$C_6D_4Cl_2$-*d*4 at room temperature.

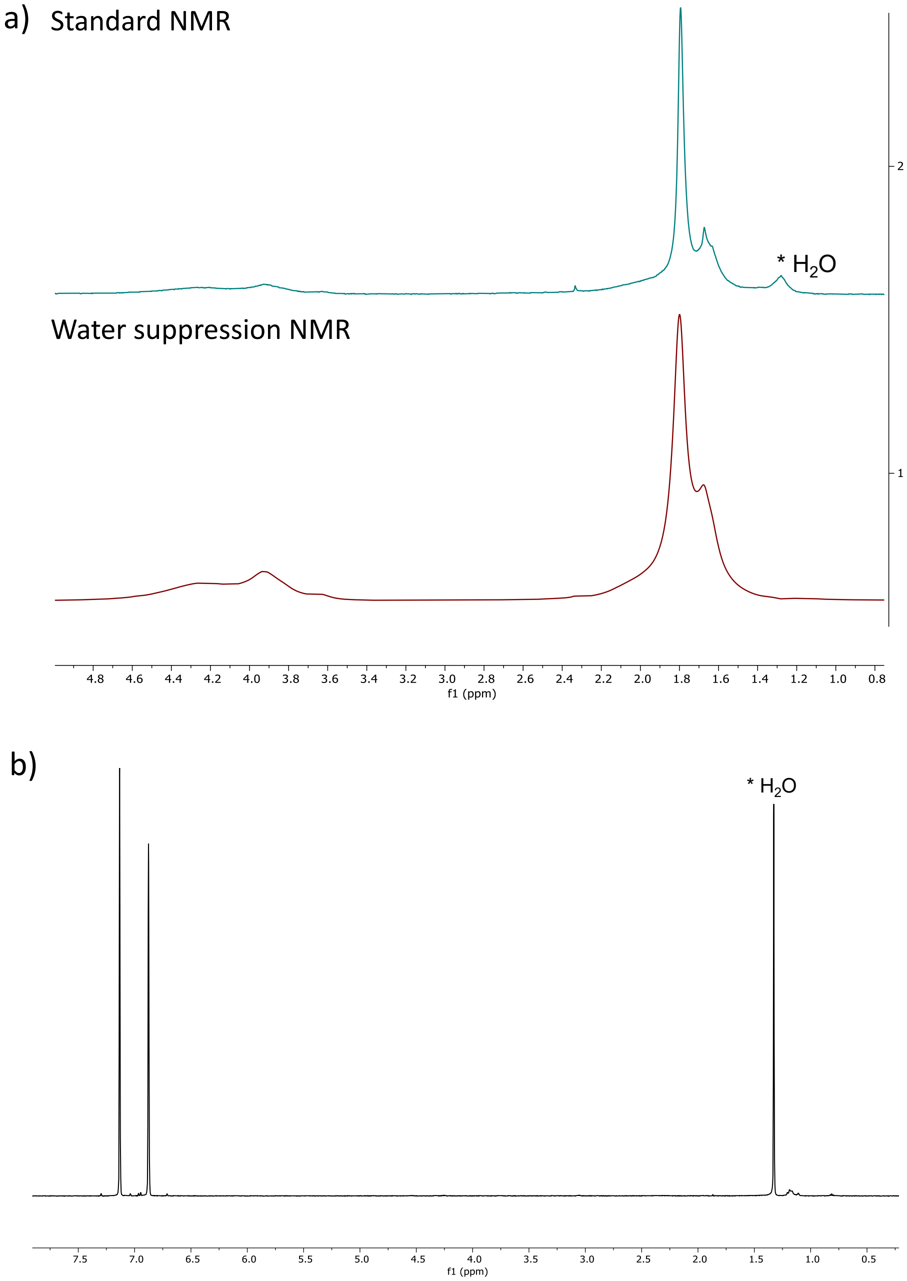

Figure S25. (a) Standard (teal curve) and water suppression (scarlet curve) ^{1}H NMR spectra acquired in o-$C_6D_4Cl_2$-*d*4 at RT showing the suppression of water peak. **(**b) ^{1}H NMR spectrum of water in o-$C_6D_4Cl_2$-*d*4 showing a peak at ~1.30 ppm.

References

1. Onwubiko, A. et al. Fused electron deficient semiconducting polymers for air stable electron transport. *Nat. Commun.* **9**, 416 (2018).

2. Marks, A., Chen, X. et al. Synthetic nuances to maximize n-type organic electrochemical transistor and thermoelectric performance in fused lactam polymers. *J. Am. Chem. Soc.* **144**, 4642-4656 (2022).

3. Alsufyani, M. et al. Lactone backbone density in rigid electron-deficient semiconducting polymers enabling high n-type organic thermoelectric performance. *Angew. Chem. Int. Ed.* **61**, e202113078 (2022).

4. Cao. Y. et al. Highly efficient NIR-II photothermal conversion based on an organic conjugated polymer, *Chem. Mater.* **29**, 718-725 (2017).

5. Lu, Y. et al. Persistent conjugated backbone and disordered lamellar packing impart polymers with efficient n-doping and high conductivities. *Adv. Mater.* **33**, 2005946 (2021).

6. Mistry, A. et al. The synthesis and STM/AFM imaging of 'olympicene' benzo[cd]pyrenes. *Chem. Eur. J.* **21**, 2011-2018 (2015).

7. Perdigão, L.M.A. LMAPper – Where scanning probe microscopy and molecular visualisation meet. *https://sourceforge.net/projects/spm-and-mol-viewer/.*

8. Moro, S. et al. The effect of glycol side chains on the assembly and microstructure of conjugated polymers. *ACS Nano* **16**, 21303–21314 (2022).

9. Lin, M. C. and Laidler, K. J. Some aspects of cis–trans-isomerization mechanisms. *Can. J. Chem.* **46**, 973 (1968).
